# SMC complexes: Lifting the lid on loop extrusion

**DOI:** 10.1016/j.ceb.2021.12.003

**Published:** 2022-02

**Authors:** Torahiko L. Higashi, Frank Uhlmann

**Affiliations:** 1 Chromosome Segregation Laboratory, The Francis Crick Institute, London, NW1 1AT, UK; 2 Department of Cellular Biochemistry, Kyushu University, Fukuoka, 812-8582, Japan

## Abstract

Loop extrusion has emerged as a prominent hypothesis for how SMC complexes shape chromosomes – single molecule *in vitro* observations have yielded fascinating images of this process. When not extruding loops, SMC complexes are known to topologically entrap one or more DNAs. Here, we review how structural insight into the SMC complex cohesin has led to a molecular framework for both activities: a Brownian ratchet motion, associated with topological DNA entry, might repeat itself to elicit loop extrusion. After contrasting alternative loop extrusion models, we explore whether topological loading or loop extrusion is more adept at explaining *in vivo* SMC complex function. SMC variants that experimentally separate topological loading from loop extrusion will in the future probe their respective contributions to chromosome biology.

## Introduction

Ring-shaped SMC complexes are universal chromosome constituents that facilitate life with genomic DNA molecules that are typically 100s–1000s times longer than the cells that harbor them [1, 2, 3]. Bacterial SMC complexes, like eukaryotic condensin, probably reflect the ancestral function of these complexes. They compact chromosomes and facilitate the topological resolution of sister chromatids during chromosome segregation. The cohesin complex is a crucial evolutionary addition in eukaryotes. It holds sister chromatids together following DNA replication, the basis for chromosome alignment on the spindle apparatus during cell divisions. Cohesin also delineates chromatin domains in the interphase nucleus and participates in DNA break repair and restart of stalled replication forks. Most eukaryotes contain a further Smc5–Smc6 complex that guards the accuracy of DNA repair by recombination.

All studied SMC complexes have been observed to topologically entrap DNA *in vivo* and/or *in vitro* [4, 5, 6, 7, 8]. In the case of cohesin, topological DNA entrapment provides a powerful means for embracing two sister chromatids [9]. Topological DNA capture could also underlie most other SMC complex functions. For instance, cohesin could establish chromatin loops in the interphase nucleus by sequentially entrapping two DNA sequences along one chromatid. Condensin could similarly form chromatin loops by sequential DNA capture during chromosome condensation. Establishment of interactions between more than one DNA by topological embrace could also assist DNA repair and recombination.

Over the last years, an alternative hypothesis for SMC complex function has gained in popularity, that of loop extrusion [3,10,11]. According to this proposal, SMC complexes form a small DNA loop that is enlarged through an active DNA translocation process. Single molecule *in vitro* experiments have provided captivating movies of cohesin and condensin engaged in loop extrusion [12, 13∗, 14∗, 15∗, 16∗∗, 17]. If seeing is believing, these observations are too good for the loop extrusion hypothesis not to be true. However, very low external forces stall loop extrusion, and the impact of a dense chromatin environment is only beginning to be understood.

## Topological DNA entry into the cohesin ring

**Figure 1:**
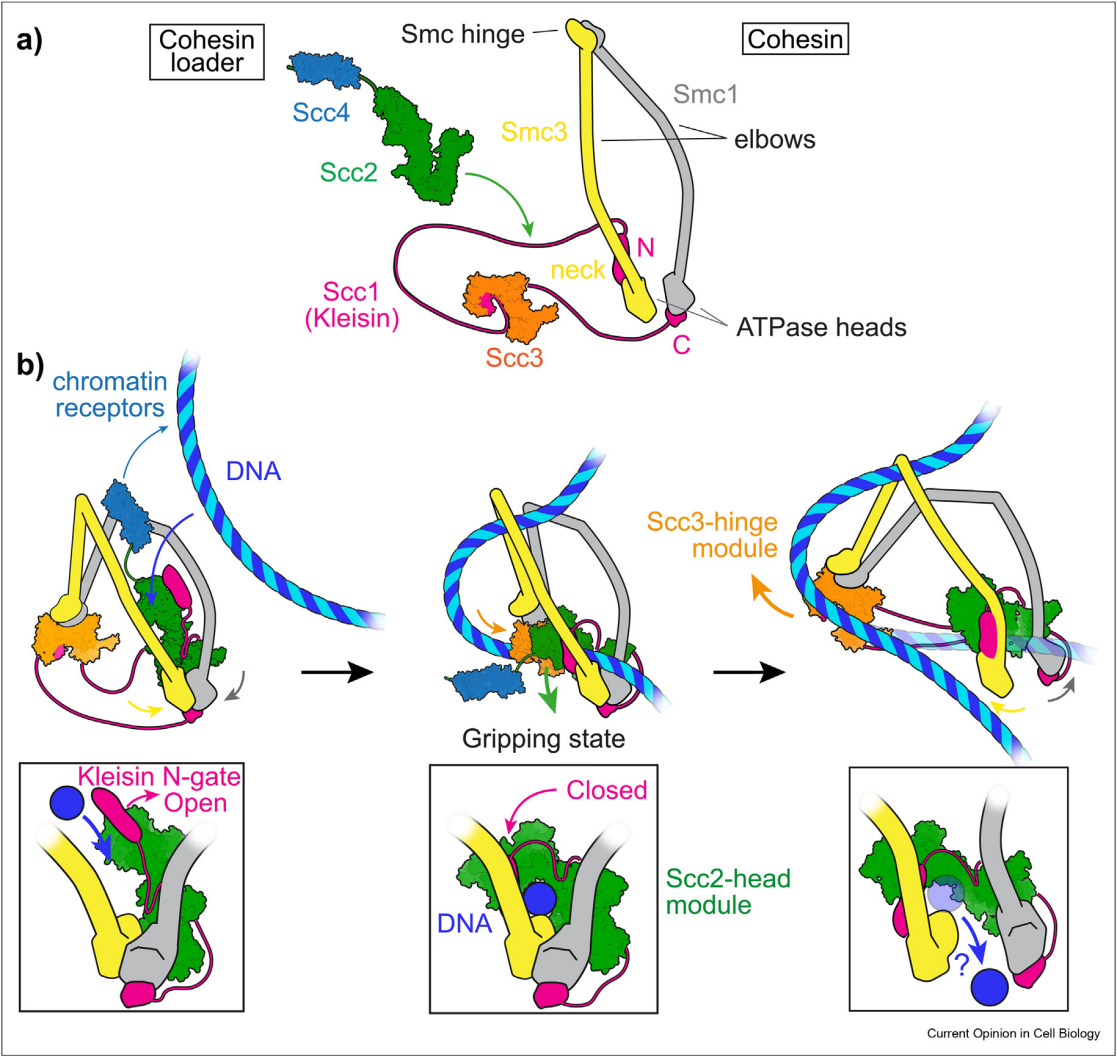
**Molecular model for topological DNA entry into the cohesin ring. a)** Structural overview of the cohesin complex components, as well as of the cohesin loader, and their assembly. **b)** Model for DNA entry into the cohesin ring through the kleisin N-gate followed, or not, by ATPase head gate passage.

Before discussing loop extrusion, we consider the mechanism of topological DNA entry into the cohesin ring. Figure 1a illustrates the cohesin complex. The SMC subunits Smc1 and Smc3 are linked at their hinge dimerization interface, from where coiled coils stretch out towards the ATPase head domains, with an inflection point at the “elbows.” The heads are bridged by a kleisin subunit whose N-terminus associates with the Smc3 “neck,” while its C-terminus binds the Smc1 head. The kleisin N- and C-termini are connected by a long and largely unstructured peptide linker. To the middle of this linker binds the HEAT-repeat subunit Scc3. The “cohesin loader,” consisting of another HEAT-repeat subunit Scc2 and the chromatin adaptor Scc4, transiently binds the kleisin unstructured region between N-terminus and Scc3 to facilitate topological loading.

Two recent cryo-EM structures of human and fission yeast cohesin have revealed how the above components come together when the complex binds to DNA [18,19]. Both structures show engaged ATPase heads, bound to a non-hydrolyzable ATP analogue. DNA is attracted to a composite positively charged surface that spans the top of both ATPase heads. The cohesin loader subunit Scc2 in turn makes extensive contacts with both the ATPase heads, as well as with the DNA. Notably, Scc2 has undergone a striking conformational change, compared to its previously observed extended form [20]. The subunit grips onto the DNA, lending its name to this “gripping state” (Figure 1b, middle). A band of positively charged surface residues on Scc2 engage the DNA [21, 22∗, 23]. Jointly, the ATPase heads and the cohesin loader thus form a tight “Scc2-head” DNA binding module. A second DNA binding module is provided by the other HEAT-repeat subunit, Scc3. In the gripping state, Scc3 docks onto the back of Scc2 and, like Scc2, binds DNA using a stretch of positive charges [24]. In addition, Scc3 also serves as a platform for the SMC hinge, which touches down following SMC coiled coil folding at the elbows. Together, Scc3 and the hinge thereby form an “Scc3-hinge” DNA binding module.

In the gripping state, DNA has already entered the cohesin ring through the interface between the kleisin N-terminus and the Smc3 neck, denoted the “kleisin N-gate.” If we model Scc2 prior to DNA binding, in its extended conformation, the likely DNA entry route emerges (Figure 1b, left). ATP-dependent head engagement initially disfavors the kleisin N-interaction with the Smc3 neck, which opens the kleisin N-gate. Several lines of evidence suggest that DNA passes through this open kleisin N-gate into the cohesin ring, including DNA–protein crosslink mass spectrometry data, FRET analyses, and cryo-EM structures of the open kleisin N-gate [19,25,26]. In the gripping state, arrival of the DNA, together with the concomitant Scc2 conformational change, result in N-gate closure.

Gripping state formation triggers ATP hydrolysis, resulting in ATPase head disengagement. Return of Scc2 to its extended conformation promotes head separation, with two consequences. First, the DNA could pass through this open “head gate,” guided by the Scc3-hinge module, which swings free from Scc2 as the latter changes conformation (Figure 1b, right) [16]. On the other hand, residual electrostatic interactions could retain DNA within the Scc2-head module. Interface crosslinking experiments with cohesin and other SMC complexes have found evidence for this configuration [27, 28, 29∗]. Secondly, and probably more importantly, head disengagement firmly locks the kleisin N-gate. While the heads are engaged, the kleisin-N/Smc3-neck interface tends to open. Therefore, ATP hydrolysis and head separation are crucial to reach stable topological DNA embrace.

**Figure 2:**
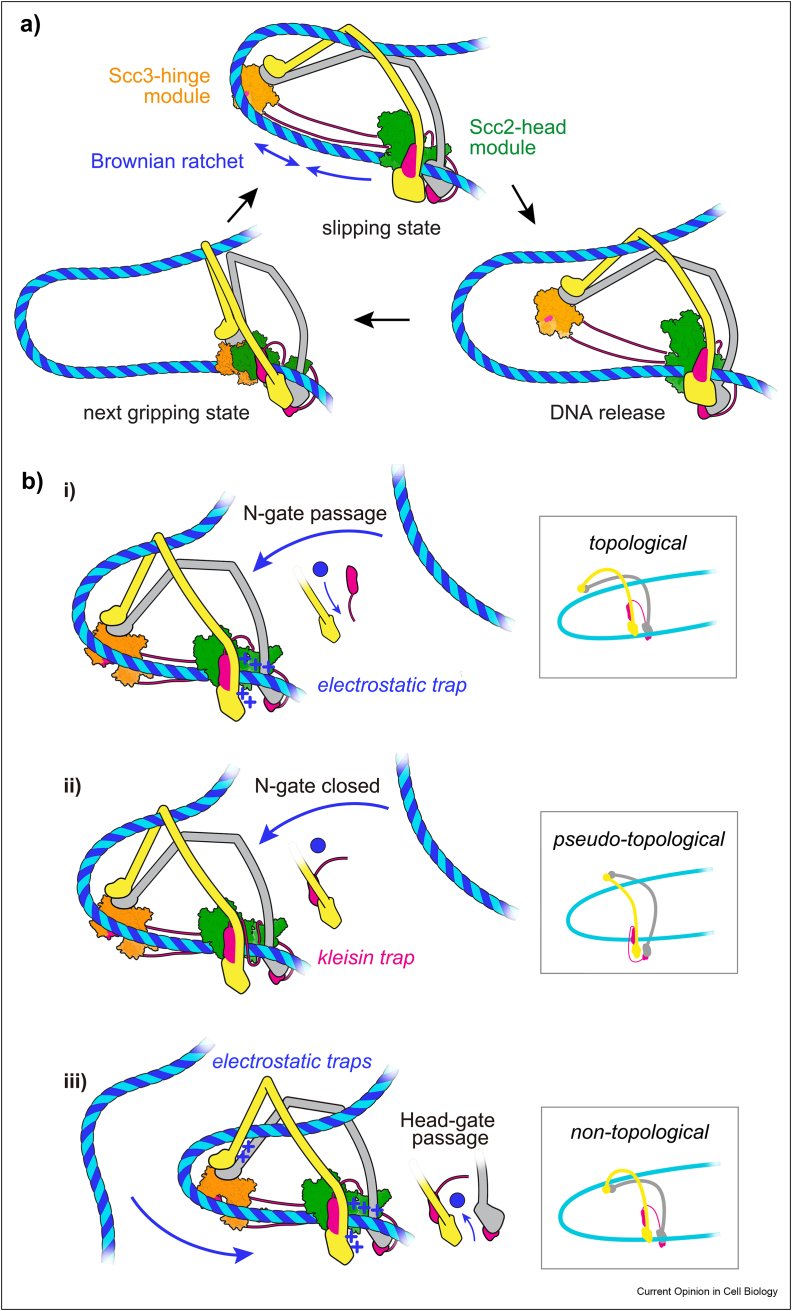
**A Brownian ratchet model for loop extrusion by cohesin. a)** Repeated cycles of gripping-to-slipping state transitions, followed by DNA release from the Scc3-hinge module, enact a ratchet that drives loop extrusion. **b)** Three scenarios of how DNA could engage with the cohesin complex during loop extrusion, resulting in a topological, pseudo-topological or non-topological interaction.

An alternative DNA entry pathway into the cohesin ring has been proposed, in which DNA arrives to the gripping state “bottom–up,” through the ATPase head gate [18,22]. In this scenario, topological DNA entry has not been achieved in the gripping state. DNA will have to enter the cohesin ring as consequence of ATP hydrolysis, potentially through the SMC hinge, which is seen as partly open in the human cohesin gripping state structure [18]. Below (Figure 2b, *iii*), we depict a possible DNA path in this scenario. The “top–down” model shown in Figure 1b has the benefit of a clearly demarcated DNA trajectory into the cohesin ring. The cohesin loader's Scc4 subunit is suitably positioned to recruit cohesin to chromatin receptors (Figure 1b, left), for example, the RSC chromatin remodeler or the Ctf19 inner kinetochore complex [30,31].

## Turning topological entry into loop extrusion

How could DNA entry into the cohesin ring turn into loop extrusion? In the ATP-bound gripping state, the Scc2-head and Scc3-hinge DNA binding modules lie juxtaposed and have introduced a DNA bend (Figure 1b, middle). Ensuing ATP hydrolysis changes the behavior of both modules. The Scc2-head module gives up its tight DNA grip, but may retain loose DNA contact that allows lateral DNA sliding (Figure 2a, “slipping state”). The Scc3-hinge module, in turn, disengages from the Scc2-head module and is free to enter a swinging motion. This motion will turn the DNA bend into a small DNA loop. Loop extrusion has begun.

The Scc3-hinge module does not change its mode of DNA interaction in the slipping state. Nevertheless, DNA binding to Scc3 outside the gripping state has a finite lifetime, probably in the millisecond range [16]. Therefore, a period follows in which DNA is released from the Scc3-hinge module (Figure 2a, DNA release). Next, the ATPase heads can re-engage in the presence of ATP and the next gripping state can assemble. This state differs from the first gripping state only in that the DNA bend is already a small loop (Figure 2a, next gripping state). ATP hydrolysis now leads to the next slipping state, during which the loop extends further.

What is the energy source for loop growth during these gripping-to-slipping state cycles? Once the Scc3-hinge module disengages from the Scc2-head module, Brownian motion can take it in only one direction, that of loop growth. Loop extrusion might thus be driven by a ***Brownian ratchet***, in which the energy from ATP binding and hydrolysis merely switches the ratchet between its states. High speed atomic force microscopy of the condensin complex revealed Brownian fluctuations of the hinge, when detached from the heads [32]. Computational simulations of loop extrusion by a Brownian ratchet show good agreement with experimental observations, particularly with the large spread of loop extrusion speeds and the small external force required to stall extrusion [16].

## The topology of loop extrusion

One can imagine three topologies in which DNA intersects with the cohesin ring during loop extrusion. In the above-described scenario, loop extrusion follows topological DNA entry into the cohesin ring. One DNA passes through the ring, while a second DNA is moved along the outside of the ring by the ratchet (Figure 2b, *i*). The second DNA retains contact with the Scc2-head module in what could be called an “electrostatic trap.”

Experiments with human cohesin suggested that DNA might not topologically enter cohesin during loop extrusion [13]. To explain this, in a second scenario, the gripping state might form without kleisin N-gate passage and therefore without topological DNA entry into the cohesin ring [19]. N-gate opening might not be hard-wired to ATPase head engagement while DNA approaches the gripping state (Figure 2b, *ii*). Following ATP hydrolysis, in this scenario, head gate passage is blocked by a “kleisin trap.” As a consequence, DNA threads in and out of the cohesin ring in a “pseudo-topological” trajectory.

Recent *in vitro* observations revealed that large DNA-bound obstacles can be bypassed by loop-extruding cohesin and condensin [33]. This is most readily explained if the transported DNA lies outside of the SMC ring, for example, in the “topological” model (Figure 2b, *i*). Obstacle bypass could alternatively be achieved by an entirely “non-topological” mode of DNA binding. In this third scenario, DNA wraps around cohesin from the outside and enters the ratchet bottom–up through the ATPase head gate (Figure 2b, *iii*). A second DNA contact point could be a positively charged patch at the SMC hinge, which has been implicated in loop extrusion [34]. The three described DNA topologies are not mutually exclusive and might be taken up by different SMC complexes with different frequencies. (As indicated above, the bottom-up gripping state configuration shown in Figure 2b, *iii* could turn into topological DNA entry if the hinge transiently opened.)

## Alternative models for DNA loop extrusion by SMC complexes

**Figure 3:**
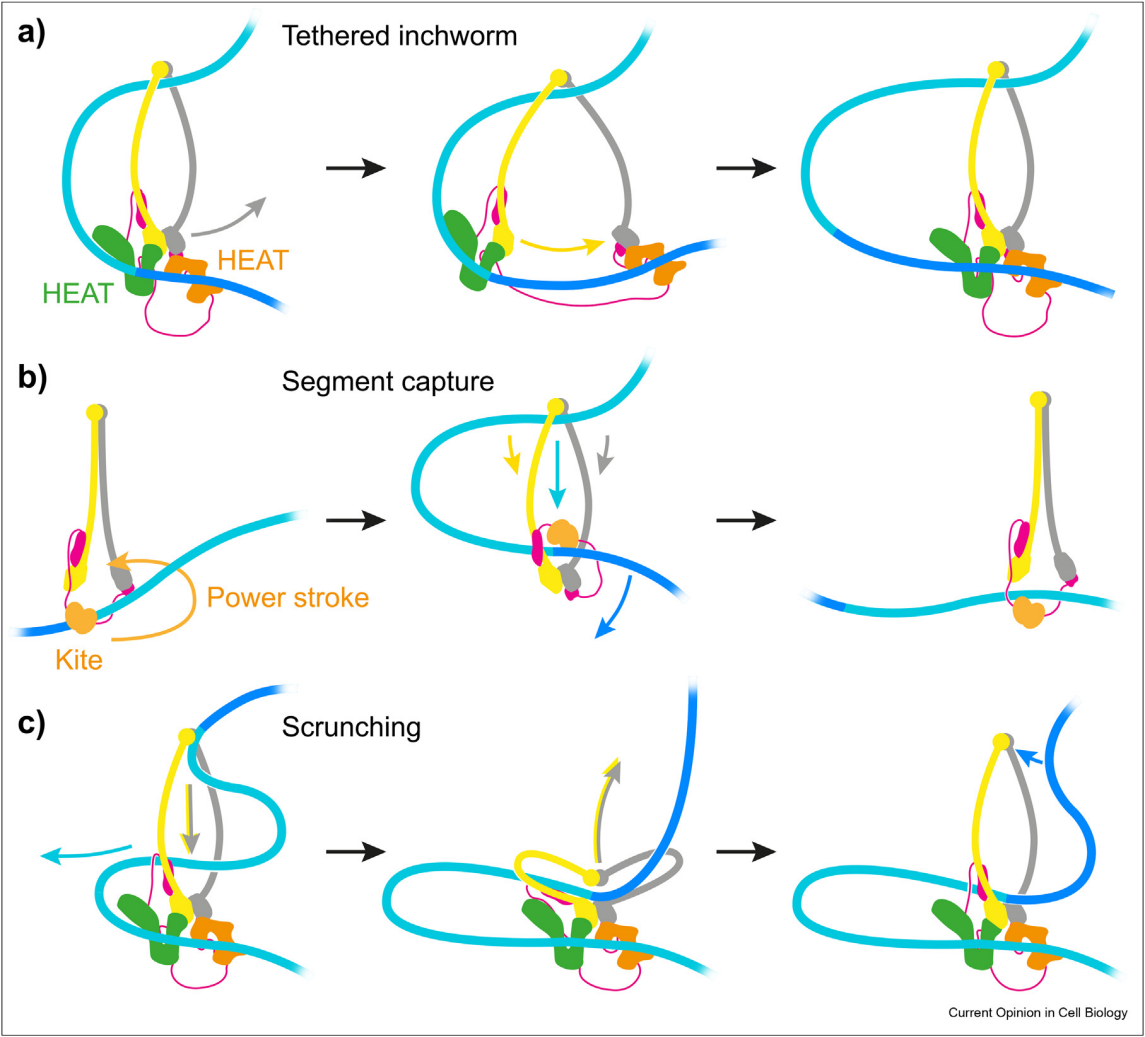
**Alternative models for loop extrusion by SMC complexes. a)** The tethered inchworm model, in which the two HEAT-repeat subunits (HEAT) step along the DNA. **b)** The segment capture model, inspired by prokaryotic SMC complexes, in which the Kite subunits (Kite) perform a power stroke to loop DNA. An additional DNA anchor (not shown) is required for loop extrusion. **c)** The scrunching model suggests that the hinge captures DNA and hands it over to the heads to enlarge the loop.

In this section, we compare the ***Brownian ratchet*** model to other proposed models for loop extrusion. The first offered model was the ***tethered inchworm*** model [35]. Formulated without the benefit of current structural knowledge, this model also features the two HEAT subunits as DNA binding elements, which perform a scissoring motion while remaining connected by a flexible kleisin linker (Figure 3a). Instead of forming head and hinge modules, the HEAT subunits associate with the two ATPase heads. DNA affinity of the HEAT subunits is postulated to alternate during the ATP hydrolysis cycle, such that the two heads step along the DNA. The model includes a topological DNA interaction. Obstacle bypass is possible while the HEAT subunits step along the outside DNA. Consistent with the idea of HEAT subunits as moving elements, loop extrusion has so far been observed with cohesin and condensin that contain HEAT subunits connected by flexible kleisins, but not with prokaryotic or Smc5–Smc6 complexes that, instead of HEAT subunits, contain two conjoined Kite DNA binding elements [36,37].

An alternative ***DNA segment capture*** model is inspired by features of prokaryotic SMC complexes [38,39]. This model makes use of one major DNA binding site, formed by the Kite subunits and SMC heads, similar to the Scc2-head module in the ratchet model. A key distinction of the segment capture model is a hypothetical power stroke by the Kite-head module (Figure 3b). This stroke swings DNA by at least 130° [40] to throw a DNA loop into the SMC ring. After the loop is formed, DNA is released from the Kite-head module while the second DNA is pushed from the hinge to the heads as the coiled coils zip up, resulting in DNA translocation. An additional DNA anchor, required to enact loop extrusion, has been postulated but not yet been identified. The segment capture model is also topological in nature, but in this case the transported DNA passes through the SMC ring. A variant segment capture model was recently proposed for eukaryotic condensin [28]. This ***hold-and-feed*** model ascribes the power stroke to condensin's Ycs4 HEAT subunit as it transitions from its extended to its gripping state conformation. As DNA binding energy is likely absorbed in this conformational transition, the nature of the power stroke remains to be ascertained.

Lastly, a ***scrunching*** model for loop extrusion is based on observed conformational transitions of the condensin complex between an open O and an ATP-bound B shape in which the SMC hinge touches the heads [32]. Like in the ratchet model, thermal fluctuations between DNA binding sites at the head and hinge form the basis for loop formation and extrusion (Figure 3c). In contrast, the scrunching model predicts DNA movement with opposite direction, being captured by the SMC hinge in the unfolded O state and released in the folded B state. This requires regulated DNA binding to hinge and heads by yet unknown mechanisms. The HEAT subunits are thought to form a static DNA anchor. The original depiction of the scrunching model involves topological entrapment of the transported DNA. A variant scrunching model, termed ***swing and clamp*** [34], is based on observations with human cohesin and postulates an opposite ATP-bound state with distant hinge and heads. Future experiments will be directed at comparing and testing predictions from these diverse models for DNA loop extrusion by SMC complexes.

## Loop extrusion and *in vivo* SMC complex function

We proposed that topological DNA entry into the cohesin ring involves conformational transitions that, if repeated, drive loop extrusion [19]. According to this view, loop extrusion is a byproduct of topological loading, manifest under *in vitro* observation conditions. Loop extrusion experiments typically involve unphysiologically low salt concentrations and intercalating dyes that make DNA thinner and more bendable [12, 13∗, 14∗, 15∗, 16∗∗, 17]. What would happen if SMC complexes attempted loop extrusion *in vivo*?

### Obstacle encounters

**Figure 4:**
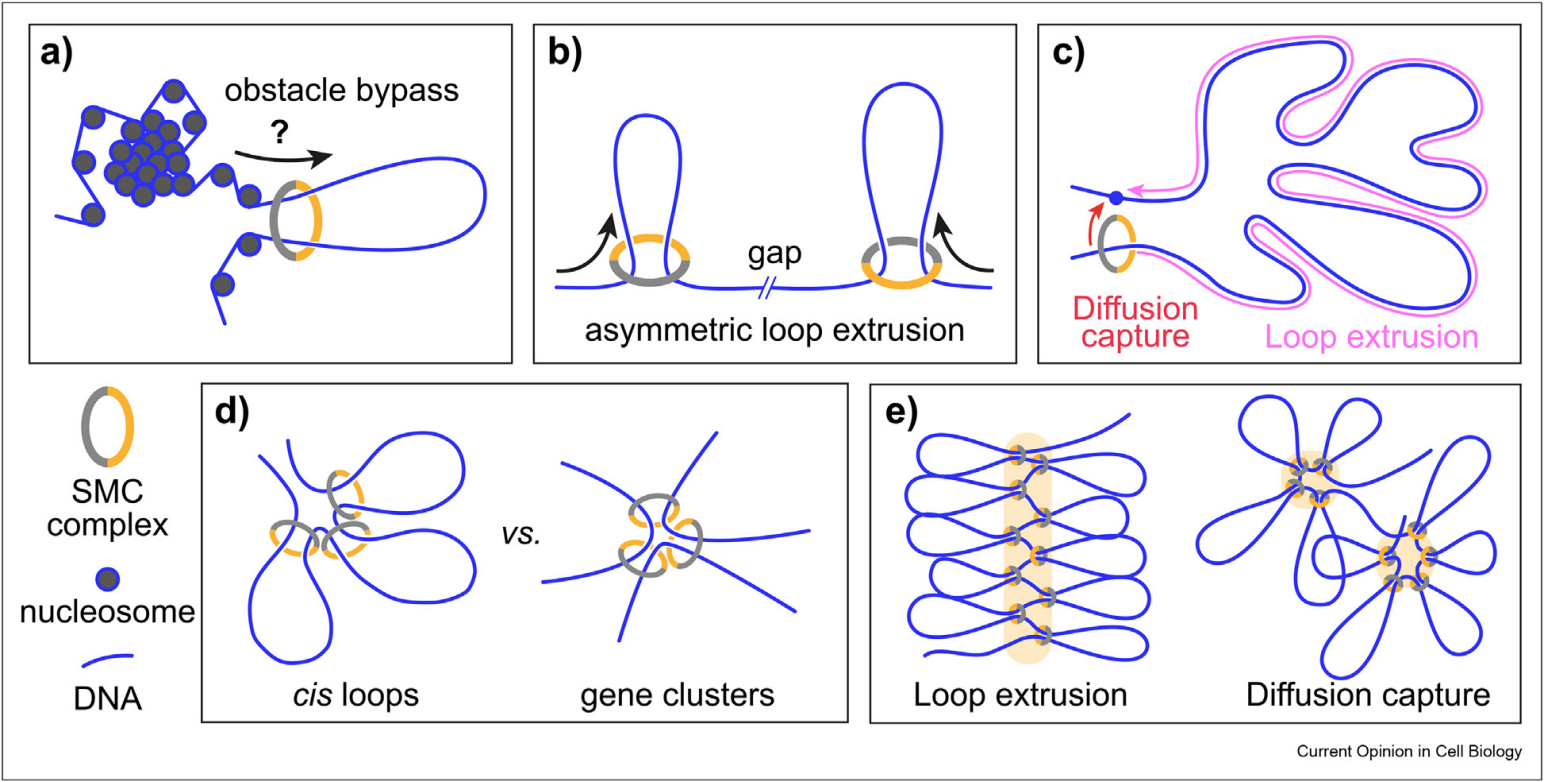
**Challenges to *in vivo* DNA loop extrusion. a)** DNA-bound obstacles and sparse free DNA must be navigated by advancing SMC complexes. **b)** Condensin extrudes DNA asymmetrically, leaving gaps during chromosome formation. **c)** Long stretches of chromatin must be extruded, where diffusion capture offers shortcuts. **d)** Loop extrusion solely generates intra-chromosomal *cis* loops, while SMC complexes also engage in inter-chromosomal interactions. **e)** Loop extrusion predicts formation of a linear SMC backbone. SMC complexes are observed to form clusters, as expected from diffusion capture.

Nucleosomes interfere with *in vitro* cohesin loading, while known *in vivo* cohesin and condensin loading sites coincide with nucleosome-free regions [30,41, 42, 43]. These observations suggest that SMC complexes require naked DNA as a substrate. This said, loop extruding SMC complexes show a remarkable ability to bypass DNA-bound obstacles, including ones much larger than the SMC ring [17,33]. As we have seen above, obstacle bypass can be explained by loop extrusion models in which the transported DNA lies outside of the ring. As yet, bypass studies have been performed using individual or sparse roadblocks, with readily available free DNA between obstacles. Free DNA is much more rarely found *in vivo* (Figure 4a), where hindrances take on much larger dimensions, for instance nucleosome clusters [44,45]. Loop extrusion in *Xenopus* egg extracts could only be observed after nucleosomes were depleted [15]. How SMC complexes behave when encountering native chromatin substrates remains an important open question.

### Gaps during chromosome condensation

Cohesin symmetrically extrudes DNA loops, while condensin is typically seen to asymmetrically reel in one of the two DNAs [12, 13∗, 14∗,^17^]. In a pseudo-topological scenario, both DNAs pass through the SMC ring. Whether the same DNA always engages with the ratchet, or both DNAs periodically take turns, could decide on asymmetric or symmetric loop growth [16]. In the case of topological or non-topological models, only one of the DNAs has access to the ratchet. The amount of friction at the second DNA contact point might influence whether extrusion occurs asymmetrically or symmetrically. Irrespective of the mechanism, simulations of asymmetric loop extrusion by condensin [46] reveal gaps that preclude completion of chromosome condensation (Figure 4b). Condensins that bypass each other to form z-loops [47] could fill these gaps; however, at a risk of creating chromatin entanglement.

### Loop formation

In higher eukaryotes, loops formed by cohesin and condensin are often megabases in size [48,49]. Are these loops easiest formed by extrusion? Alternatively, loops could form when SMC complexes sequentially topologically entrap two loop anchor sequences in a process termed “diffusion capture” (Figure 4c) [50,51]. Cohesin can sequentially entrap two DNAs [52], whether condensin shares this ability will be important to establish. Cohesin depends on the cohesin loader for loop extrusion, found at nucleosome-free gene promoters [41,53, 54, 55]. Following topological loading, the cohesin loader is replaced by Pds5, a HEAT subunit that shares structural similarity with Scc2 [56] but attenuates rather than stimulates cohesin's ATPase and is therefore unlikely to support loop extrusion [21,57]. At loop anchor sites, cohesin appears to have arrived with Pds5 [55,58]. RNA polymerases that push Pds5-containing cohesin along chromosomes, following diffusion capture, could offer an alternative explanation for the observed looping pattern [59,60]. Finally, budding yeast chromosomes display cohesin-dependent chromatin loops, even though budding yeast cohesin has so far been refractory to demonstrating loop extrusion. Bridging-induced phase separation of cohesin and DNA, a form of diffusion capture, has been suggested as an alternative looping mechanism [61∗, 62∗, 63∗].

### cis Loops vs. gene clusters

A perceived benefit of loop extrusion is that it provides a fool-proof mechanism for interactions within a chromatin chain (*cis* loops), rather than between neighboring chromatin chains or chromosomes (Figure 4d). However, cohesin is of course best known for establishing interactions between two sister chromatids. Experimental observations suggest that also condensin promotes sister chromatid interactions [64], as well as forms tRNA and histone gene clusters involving more than one chromosome [65, 66, 67, 68, 69]. Thus, SMC complexes do engage in trans chromatin interactions that loop extrusion is unable to explain. Excluded volume interactions and entropy-driven forces ensure that even diffusion capture interactions maintain a preference for intra-chain interactions [70,71], making it an attractive explanation for favored, yet non-exclusive, *cis* loop formation.

### Mitotic chromosome formation

Computational simulations of loop extrusion have demonstrated its ability to generate mitotic chromosomes with desirable properties [11], a linear condensin backbone from where DNA loops emerge that repel neighboring chromatids to achieve chromosome individualization (Figure 4e). Closer inspection of loop extrusion simulations, run alongside simulations of chromosome formation by diffusion capture [51], revealed limitations of the loop extrusion mechanism. Diffusion capture more readily compacts chromatin and shortens chromosome axes, hallmarks of mitotic chromosome condensation. Diffusion capture, but not loop extrusion, also replicates the observed reduced mitotic chromatin mobility. Finally, superresolution microscopy revealed that condensin forms clusters within both yeast and human mitotic chromosomes [51,72], similar to those generated in diffusion capture simulations, but distinct from the linear condensin arrays predicted to result from loop extrusion.

## Outlook

The largely thermal forces that SMC complexes deploy to move DNA raise the question whether loop extrusion is a potent genome organizing principle. In addition to moving by themselves, topologically loaded SMC complexes are pushed by RNA polymerases, strong external translocases. The possible role of transcription in shaping SMC complex-dependent genome architecture deserves further attention. Importantly, SMC variants can now be sought that separate topological loading and loop extrusion [73]. For example, yeast condensin's Ycg1 subunit plays a critical role in loop extrusion, while vertebrate condensin lacking its XCAP-G ortholog retains the ability to shape and individualize chromosomes [32,74]. The molecular models discussed in this review should aid the rational design of SMC variants that separate topological loading from loop extrusion. This will clarify the respective contributions of the two activities to genome organization and stability.

## Conflict of interest statement

The authors declare no conflict of interest.

## References

[1] Hirano T. (2016). Condensin-based chromosome organization from bacteria to vertebrates. Cell.

[2] Uhlmann F. (2016). SMC complexes, from DNA to chromosomes. Nat Rev Mol Cell Biol.

[3] Yatskevich S., Rhodes J., Nasmyth K. (2019). Organization of chromosomal DNA by SMC complexes. Annu Rev Genet.

[4] Gruber S., Haering C.H., Nasmyth K. (2003). Chromosomal cohesin forms a ring. Cell.

[5] Cuylen S., Metz J., Haering C.H. (2011). Condensin structures chromosomal DNA through topological links. Nat Struct Mol Biol.

[6] Murayama Y., Uhlmann F. (2014). Biochemical reconstitution of topological DNA binding by the cohesin ring. Nature.

[7] Kanno T., Berta D.G., Sjögren C. (2015). The Smc5/6 complex is an ATP-dependent intermolecular DNA linker. Cell Rep.

[8] Niki H., Yano K. (2016). In vitro topological loading of bacterial condesin MukB on DNA, preferentially single-stranded DNA, rather than double-stranded DNA. Sci Rep.

[9] Haering C.H., Farcas A.M., Arumugam P., Metson J., Nasmyth K. (2008). The cohesin ring concatenates sister DNA molecules. Nature.

[10] Alipour E., Marko J.F. (2012). Self-organization of domain structures by DNA-loop-extruding enzymes. Nucleic Acids Res.

[11] Goloborodko A., Imakaev M., Marko J.F., Mirny L. (2016). Compaction and segregation of sister chromatids via active loop extrusion. Elife.

[12] Ganji M., Shaltiel I.A., Bisht S., Kim E., Kalichava A., Haering C.H., Dekker C. (2018). Real-time imaging of DNA loop extrusion by condensin. Science.

[bib13] Davidson I.F., Bauer B., Goetz D., Tang W., Wutz G., Peters J.-M. (2019). DNA loop extrusion by human cohesin. Science.

[bib14] Kim Y., Shi Z., Zhang H., Finkelstein I.J., Yu H. (2019). Human cohesin compacts DNA by loop extrusion. Science.

[bib15] Golfier S., Quail T., Kimura H., Brugués J. (2020). Cohesin and condensin extrude DNA loops in a cell cycle-dependent manner. Elife.

[bib16] Higashi T.L., Pobegalov G., Tang M., Molodtsov M.I., Uhlmann F. (2021). A Brownian ratchet model for DNA loop extrusion by the cohesin complex. Elife.

[17] Kong M., Cutts E.E., Pan D., Beuron F., Kaliyappan T., Xue C., Morris E.P., Musacchio A., Vannini A., Greene E.C. (2020). Human condensin I and II drive extensive ATP-dependent compaction of nucleosome-bound DNA. Mol Cell.

[bib18] Shi Z., Gao H., Bai X., Yu H. (2020). Cryo-EM structure of the human cohesin-NIPBL-DNA complex. Science.

[bib19] Higashi T.L., Eickhoff P., Sousa J.S., Locke J., Nans A., Flynn H.R., Snijders A.P., Papageorgiou G., O'Reilly N., Chen Z.A. (2020). A structure-based mechanism for DNA entry into the cohesin ring. Mol Cell.

[20] Kikuchi S., Borek D.M., Otwinowski Z., Tomchick D.R., Yu H. (2016). Crystal structure of the cohesin loader Scc2 and insight into cohesinopathy. Proc Natl Acad Sci USA.

[21] Murayama Y., Uhlmann F. (2015). DNA entry into and exit out of the cohesin ring by an interlocking gate mechanism. Cell.

[bib22] Collier J.E., Lee B.-G., Roig M.B., Yatskevich S., Petela N.J., Metson J., Voulgaris M., Gonzalez Llamazares A., Löwe J., Nasmyth K.A. (2020). Transport of DNA within cohesin involves clamping on top of engaged heads by Scc2 and entrapment within the ring by Scc3. Elife.

[23] Kurokawa Y., Murayama Y. (2020). **DNA binding by the Mis4**^**Scc2**^**loader promotes topological DNA entrapment by the cohesin ring**. Cell Rep.

[24] Hara K., Zheng G., Qu Q., Liu H., Ouyang Z., Chen Z., Tomchick D.R., Yu H. (2014). Structure of cohesin subcomplex pinpoints direct shugoshin-Wapl antagonism in centromeric cohesion. Nat Struct Mol Biol.

[bib25] Muir K.W., Li Y., Weis F., Panne D. (2020). The structure of the cohesin ATPase elucidates the mechanism of SMC-kleisin ring opening. Nat Struct Mol Biol.

[bib26] Lee B.G., Merkel F., Allegretti M., Hassler M., Cawood C., Lecomte L., O'Reilly F.J., Sinn L.R., Gutierrez-Escribano P., Kschonsak M. (2020). Cryo-EM structures of holo condensin reveal a subunit flip-flop mechanism. Nat Struct Mol Biol.

[27] Chapard C., Jones R., van Oepen T., Scheinost J.C., Nasmyth K. (2019). Sister DNA entrapment between juxtaposed Smc heads and kleisin of the cohesin complex. Mol Cell.

[28] Shaltiel I.A., Datta S., Lecomte L., Hassler M., Kschonsak M., Bravo S., Stober C., Eustermann S., Haering C.H. (2021). A hold-and-feed mechanism drives directional DNA loop extrusion by condensin. BioRxiv.

[bib29] Bürmann F., Funke L.F.H., Chin J.W., Löwe J. (2021). Cryo-EM structure of MukBEF reveals DNA loop entrapment at chromosomal unloading sites. Mol Cell.

[30] Muñoz S., Minamino M., Casas-Delucchi C.S., Patel H., Uhlmann F. (2019). A role of chromatin remodeling in cohesin loading onto chromosomes. Mol Cell.

[31] Hinshaw S.M., Makrantoni V., Harrison S.C., Marston A.L. (2017). The kinetochore receptor for the cohesin loading complex. Cell.

[bib32] Ryu J.-K., Katan A.J., van der Sluis E.O., Wisse T., de Groot R., Haering C.H., Dekker C. (2020). The condensin holocomplex cycles dynamically between open and collapsed states. Nat Struct Mol Biol.

[33] Pradhan B., Barth R., Kim E., Davidson I.F., Bauer B., van Laar T., Yang W., Ryu J.-K., van der Torre J., Peters J.-M. (2021). SMC complexes can traverse physical roadblocks bigger than their ring size. BioRxiv.

[34] Bauer B.W., Davidson I.F., Canena D., Wutz G., Tang W., Litos G., Horn S., Hinterdorfer P., Peters J.-M. (2021). Cohesin mediates DNA loop extrusion by a “swing and clamp” mechanism. Cell.

[35] Nichols M.H., Corces V.G. (2018). A tethered-inchworm model of SMC DNA translocation. Nat Struct Mol Biol.

[36] Woo J.S., Lim J.H., Shin H.C., Suh M.K., Ku B., Lee K.H., Joo K., Robinson H., Lee J., Park S.Y. (2009). Structural studies of a bacterial condensin complex reveal ATP-dependent disruption of intersubunit interactions. Cell.

[bib37] Jo A., Li S., Shin J.W., Zhao X., Cho Y. (2021). Structure basis for shaping the Nse4 protein by the Nse1 and Nse3 dimer within the Smc5/6 complex. J Mol Biol.

[38] Diebold-Durand M.-L., Lee H., Ruiz Avila L.B., Noh H., Shin H.-C., Im H., Bock F.P., Bürmann F., Durand A., Basfeld A. (2017). Structure of full-length SMC and rearrangements required for chromosome organization. Mol Cell.

[bib39] Marko J.F., De Los Rios P., Barducci A., Gruber S. (2019). DNA-segment-capture model for loop extrusion by structural maintenance of chromosomes (SMC) protein complexes. Nucleic Acids Res.

[40] Nomidis S.K., Carlon E., Gruber S., Marko J.F. (2021). DNA tension-modulated translocation and loop extrusion by SMC complexes revealed by molecular dynamics simulations. BioRxiv.

[41] Lopez-Serra L., Kelly G., Patel H., Stewart A., Uhlmann F. (2014). The Scc2-Scc4 complex acts in sister chromatid cohesion and transcriptional regulation by maintaining nucleosome-free regions. Nat Genet.

[42] Toselli-Mollereau E., Robellet X., Fauque L., Lemaire S., Schiklenk C., Klein C., Hocquet C., Legros P., N'Guyen L., Mouillard L. (2016). Nucleosome eviction in mitosis assists condensin loading and chromosome condensation. EMBO J.

[43] Kim J.H., Zhang T., Wong N.C., Davidson N., Maksimovic J., Oshlack A., Earnshaw W.C., Kalitsis P., Hudson D.F. (2013). Condensin I associates with structural and gene regulatory regions in vertebrate chromosomes. Nat Commun.

[44] Nozaki T., Imai R., Tanbo M., Nagashima R., Tamura S., Tani T., Joti Y., Tomita M., Hibino K., Kanemaki M.T. (2017). Dynamic organization of chromatin domains revealed by super-resolution live-cell imaging. Mol Cell.

[45] Xu J., Ma H., Jin J., Uttam S., Fu R., Huang Y., Liu Y. (2018). Super-resolution imaging of higher-order chromatin structures at different epigenomic states in single mammalian cells. Cell Rep.

[bib46] Banigan E.J., van den Berg A.A., Brandão H.B., Marko J.F., Mirny L.A. (2020). Chromosome organization by one-sided and two-sided loop extrusion. Elife.

[47] Kim E., Kerssemakers J., Shaltiel I.A., Haering C.H., Dekker C. (2020). DNA-loop extruding condensin complexes can traverse one another. Nature.

[48] Rao S.S.P., Huang S.C., Glenn St Hilaire B., Engreitz J.M., Perez E.M., Kieffer-Kwon K.-R., Sanborn A.L., Johnstone S.E., Bascom G.D., Bochkov I.D. (2017). Cohesin loss eliminates all loop domains. Cell.

[49] Gibeus J.H., Samejima K., Goloborodko A., Samejima I., Naumova N., Nuebler J., Kanemaki M.T., Xie L., Paulson J.R., Earnshaw W.C. (2018). A pathway for mitotic chromosome formation. Science.

[50] Cheng T.M.K., Heeger S., Chaleil R.A.G., Matthews N., Stewart A., Wright J., Lim C., Bates P.A., Uhlmann F. (2015). A simple biophysical model emulates budding yeast chromosome condensation. Elife.

[bib51] Gerguri T., Fu X., Kakui Y., Khatri B.S., Barrington C., Bates P.A., Uhlmann F. (2021). Comparison of loop extrusion and diffusion capture as mitotic chromosome formation pathways in fission yeast. Nucleic Acids Res.

[52] Murayama Y., Samora C.P., Kurokawa Y., Iwasaki H., Uhlmann F. (2018). Establishment of DNA-DNA interactions by the cohesin ring. Cell.

[53] Kagey M.H., Newman J.J., Bilodeau S., Zhan Y., Orlando D.A., van Berkum N.L., Ebmeier C.C., Goossens J., Rahl P.B., Levine S.S. (2010). Mediator and cohesin connect gene expression and chromatin architecture. Nature.

[54] Zuin J., Franke V., van Ijcken W.F.J., van der Sloot A., Krantz I.D., van der Reijden M.I.J.A., Nakato R., Lenhard B., Wendt K.S. (2014). A cohesin-independent role for NIPBL at promoters provides insight in CdLS. PLoS Genet.

[55] Lengronne A., Katou Y., Mori S., Yokobayashi S., Kelly G.P., Itoh T., Watanabe Y., Shirahige K., Uhlmann F. (2004). Cohesin relocation from sites of chromosomal loading to places of convergent transcription. Nature.

[56] Lee B.-G., Roig M.B., Jansma M., Petela N., Metson J., Nasmyth K., Löwe J. (2016). Crystal structure of the cohesin gatekeeper Pds5 and in complex with kleisin Scc1. Cell Rep.

[57] Petela N.J., Gligoris T.G., Metson J., Lee B.G., Voulgaris M., Hu B., Kikuchi S., Chapard C., Chen W., Rajendra E. (2018). Scc2 is a potent activator of cohesin's ATPase that promotes loading by binding Scc1 without Pds5. Mol Cell.

[58] Schmidt C.K., Brookes N., Uhlmann F. (2009). Conserved features of cohesin binding along fission yeast chromosomes. Genome Biol.

[59] Ocampo-Hafalla M., Munoz S., Samora C.P., Uhlmann F. (2016). Evidence for cohesin sliding along budding yeast chromosomes. Open Biol.

[60] Davidson I.F., Goetz D., Zaczek M.P., Molodtsov M.I., Huis In 't Veld P.J., Weissmann F., Litos G., Cisneros D.A., Ocampo-Hafalla M., Ladurner R. (2016). Rapid movement and transcriptional re-localization of human cohesin on DNA. EMBO J.

[bib61] Costantino L., Hsieh T.-H., Lamothe R., Darzacq X., Koshland D. (2020). Cohesin residency determines chromatin loop patterns. Elife.

[bib62] Schalbetter S.A., Fudenberg G., Baxter J., Pollard K.S., Neale M.J. (2019). **Principles of meiotic chromosome assembly revealed in *S. cerevisiae***. Nat Commun.

[bib63] Ryu J.-K., Bouchoux C., Liu H.W., Kim E., Minamino M., de Groot R., Katan A.J., Bonato A., Marenduzzo D., Michieletto D. (2020). Bridging-induced phase separation induced by cohesin SMC protein complexes. Sci Adv.

[64] Lam W.W., Peterson E.A., Yeung M., Lavoie B.D. (2006). Condensin is required for chromosome arm cohesion during mitosis. Genes Dev.

[65] D'Ambrosio C., Schmidt C.K., Katou Y., Kelly G., Itoh T., Shirahige K., Uhlmann F. (2008). **Identification of *cis*-acting sites for condensin loading onto budding yeast chromosomes**. Genes Dev.

[66] Haeusler R.A., Pratt-Hyatt M., Good P.D., Gipson T.A., Engelke D.R. (2008). Clustering of yeast tRNA genes is mediated by specific association of condensin with tRNA gene transcription complexes. Genes Dev.

[67] Iwasaki O., Tanaka A., Tanizawa H., Grewal S.I.S., Noma K. (2010). Centromeric localization of dispersed Pol III genes in fission yeast. Mol Biol Cell.

[68] Yuen K.C., Slaughter B.D., Gerton J.L. (2017). Condensin II is anchored by TFIIIC adn H3K4me3 in the mammalian genome and supports the expression of active dense gene clusters. Sci Adv.

[69] Paul M.R., Markowitz T.E., Hochwagen A., Ercan S. (2018). **Condensin depletion causes genome decompaction without altering the level of global gene expression in *Saccharomyces cerevisiae***. Genetics.

[70] Dockhorn R., Sommer J.-U. (2011). A model for segregation of chromatin after replication: segregation of identical flexible chains in solution. Biophys J.

[71] Jun S., Mulder B. (2006). Entropy-driven spatial organization of highly confined polymers: lessons for the bacterial chromosome. Proc Natl Acad Sci USA.

[72] Walther N., Hossain M.J., Politi A.Z., Koch B., Kueblbeck M., Ødegård-Fougner Ø., Lampe M., Ellenberg J. (2018). A quantitative map of human Condensins provides new insights into mitotic chromosome architecture. J Cell Biol.

[73] Sakata R., Niwa K., Ugarte La Torre D., Gu C., Tahara E., Takada S., Nishiyama T. (2021). Opening of cohesin's SMC ring is essential for timely DNA replication and DNA loop formation. Cell Rep.

[74] Kinoshita K., Kobayashi T.J., Hirano T. (2015). Balancing acts of two HEAT subunits of condensin I support dynamic assembly of chromosome axes. Dev Cell.

